# Association of Serum Calcium Levels of Preterm Neonates at Birth with Calcium Intake from Foods and Supplements by Bedridden Women during Pregnancy

**DOI:** 10.3390/healthcare12060693

**Published:** 2024-03-20

**Authors:** Aristea Gioxari, Panos Papandreou, Efstratia Daskalou, Andriana C. Kaliora, Maria Skouroliakou

**Affiliations:** 1 Department of Nutritional Science and Dietetics, School of Health Sciences, 24100 Kalamata, Greece; a.gioxari@uop.gr; 2 Department of Nutrition, IASO Hospital, 37-39 Kifissias Ave., 15123 Athens, Greece; 3 Department of Nutrition, General Hospital of Thessaloniki “G. Gennimatas”, 41 E. Amynis Ave., 54635 Thessaloniki, Greece; efidask@yahoo.gr; 4 Department of Dietetics and Nutritional Science, School of Health Science and Education, Harokopio University, 70 El. Venizelou Ave., 17676 Athens, Greece; mskour@hua.gr

**Keywords:** dietary calcium, preterm neonates, pregnancy, bed rest, calcium supplement, vitamin D

## Abstract

Bone calcium turnover is aggravated in pregnant women recommended to bed rest. In the present cross-sectional study, we aimed to clarify whether preterm neonates would benefit from calcium supplementation during pregnancy. Forty-two mothers (37.5 ± 6.7 years), recommended bed rest at home, and 42 preterm neonates (24–37 weeks gestational age) were enrolled. Neonates’ serum calcium was quantified at birth. Mothers’ calcium intake from foods and supplements during pregnancy was assessed. Serum 25-OH-D was measured in both mothers and neonates at birth. Results showed that mothers’ calcium intake from foods was significantly lower than the recommended daily reference value (*p* < 0.001), while total calcium intake including supplements was close to the calcium reference value of 1000 mg/day (*p* = 0.648). Neonates’ serum calcium concentration was significantly higher in mothers receiving calcium supplementation during pregnancy compared to mothers who did not (*p* < 0.001). A significant association between neonates’ serum calcium levels and mothers’ calcium supplementation was evident, even when adjusted to mothers’ age, pre-pregnancy BMI, gestational age, and neonates’ birth weight (beta = +0.460, *p* = 0.025). A statistically significant correlation between neonates’ and mothers’ serum 25-OH-D levels was found (r = 0.891, *p* < 0.001). In conclusion, calcium status in preterm neonates, born by bedridden women, could be enhanced after calcium supplementation during gestation.

## 1. Introduction

It is now well-confirmed that nutritional status including body weight status, before and during gestation, determines pregnancy outcomes, development of the fetus, and growth of the newborn [1]. Maternal nutritional status, which primarily depends on the mother’s diet, affects the quality and quantity of transferred nutrients to the growing fetus within a complex “supply line” [2]. At the same time, metabolic and endocrine responses of the mother, together with the associated alterations in nutrient metabolism may influence the delivery of nutrients [3].

There is a high requirement for calcium intake during pregnancy aiming to maintain maternal calcium balance and bone density, and to meet the demands of the growing fetus [4]. During gestation, calcium is transported across the placenta by an active transport process and is crucial for the growing fetus [5]. In the fetus and the neonate, regulation of calcium metabolism differs from that in the older infant and reflects the unique physiological challenges that occur during the crucial periods of growth, development, and perinatal transition [5].

In mothers, calcium absorption by the gut endothelial cells is enhanced during the second and the third trimesters of pregnancy [6]. However, bone turnover is also elevated, especially at the third semester, as evidenced by increased bone resorption markers, e.g., type I collagen C-telopeptides (CTX) and a crosslinked peptide of the carboxy-terminal telopeptide of type I collagen (ICTP) [7]. If calcium in the mothers’ diet is insufficient to meet this extra request, bone loss from the maternal skeleton is linked to a considerable risk of decreased fetal growth and bone mineralization, as well as impaired breast-milk calcium secretion [8]. According to a longitudinal study, pregnant women consuming low-calcium diets (<500 mg/d) had higher rates of bone resorption, while net balance in bone calcium turnover was positively associated with dietary calcium [9]. In a recent study by Cullers and co-workers, prenatal calcium supplementation with 1000 mg Ca/day was associated with improved bone recovery postpartum in mothers, as indicated by radial total and tibial cortical bone mass density compared to placebo controls [10].

With regard to the fetus, bone mineralization does not have a constant rate, while the greatest maternal–fetal calcium transfer occurs during the third trimester [11]. Based on a randomized controlled trial, total body bone mineral content was significantly greater in infants from calcium-supplemented mothers than from mothers receiving placebo [12]. Similarly, the fetus is dependent on the mother’s status of vitamin D, a key molecule in calcium metabolism and bone development. Vitamin D passes through the placenta and vitamin D levels in the cord represent 50–60% of maternal blood levels [13].

Premature neonates, especially those born at <28 weeks of gestation, are at a great risk of bone mineral content loss and developing metabolic bone diseases [14]. It has been reported that 55% of preterm infants with extremely low birth weight (≤1000 g) and 23% of infants with very low birth weight (<1500 and >1000 g) have metabolic bone diseases [15]. Metabolic bone disease is a significant multifactorial comorbidity resulting from inadequate calcium stores [14]. Symptomatology comprises postnatal growth failure, rickets, osteomalacia, osteoporosis, and fractures later in life [8,14,15]. Activity restriction including bed rest, at hospital or home, is still a commonly recommended treatment for pregnant women at a high risk of preterm birth [16]. However, there is evidence that prolonged bed rest is associated with enhanced bone resorption and subtle bone formation [17], which implies a further rise in maternal bone turnover.

Serum calcium concentration in the newborn is associated with various factors including vitamin D status and maternal dietary intake [18]. However, in the case of preterm neonates from bedridden mothers, the association between mothers’ nutritional status and neonates’ serum calcium levels are rather scarce. Therefore, the aim of the present study was to investigate the association between serum calcium levels of premature neonates at the first day of life and calcium intake during pregnancy by bedridden mothers. More specifically, we explored neonates’ calcium status upon mothers’ calcium supplementation and mothers’ pre-pregnancy weight status (normal weight vs. overweight/obesity).

## 2. Materials and Methods

### 2.1. Ethics Approval

The Scientific and Ethical Committee of IASO hospital approved the study protocol (approval code: #355/15218). The trial was performed according to the principles of the Helsinki Declaration and was in line with the terms of Good Clinical Practice. A written informed consent was delivered to each recruited mother provided and each mother kept a copy of the signed consent.

### 2.2. Participants and Study Design

The present work is a cross-sectional study, in which mothers and their preterm neonates were enrolled. Pregnant women who were outpatients of the IASO hospital and were recommended to bed rest due to the risk of preterm birth, were invited via written announcements (posters) and social media posts to take part in the study. Women who agreed to participate were provided with a detailed information leaflet describing the aims, methods, benefits, and potential hazards of the study.

Inclusion criteria were set as follows:

(a) Women instructed to bed rest within their home for at least two months before delivery (according to the physician’s recommendations) due to multiple gestation, vaginal bleeding, short cervical length, placenta abruption, and placenta previa. Bed rest was defined as limited ambulation of not more than 2–3 h per day with bathroom use.
(b) Preterm neonates (24–37 weeks gestational age) transferred to the Neonatal Intensive Care Unit (NICU) of the hospital.

Exclusion criteria were as follows:

(a) Women suffering from chronic diseases, e.g., inflammatory bowel diseases, irritable bowel syndrome, chronic kidney disease, malabsorption, chronic hypertension, or parathyroid diseases;
(b) Women under medication treatment that could affect serum calcium levels at least for 12 months prior to delivery;
(c) Pregnancy or neonate medical conditions, i.e., congenital anomalies, metabolic disorders, birth injuries, intrauterine infection, perinatal asphyxia, and life-threatening conditions;
(d) Refusal to consent.

### 2.3. Enrolled Mothers

An experienced dietitian conducted face-to-face interviews with the enrolled mothers. Demographic characteristics (age, duration and reasons for bed rest, and smoking habits) and anthropometric data, namely height, body weight (BW) at the day of delivery, post labor BW, pre-pregnancy BW, and weight gain during pregnancy, were collected. BW was measured to the nearest 0.1 kg with a flat scale. Height was measured with a standard stadiometer to the nearest millimeter (Seca Mode 220, Hamburg, Germany). Both measurements were performed twice and the mean values were calculated, respectively.

Pre-pregnancy body mass index (BMI) was calculated as the ratio of weight (kg) to the square of height (m^2^) and was defined as underweight, normal weight, overweight, or obese according to the “WHO, Global Database on Body Mass Index (BMI)” for adults [19]. Weight gain during pregnancy was classified as excessive or inadequate depending on pre-pregnancy BMI [20]. According to the Institute of Medicine (IOM) and the National Research Council (NRC), underweight women (BMI < 18.5 kg/m^2^) should gain 12.5 to 18 kg; normal weight women (BMI 18.5–24.9 kg/m^2^) 11.5 to 16 kg; women with overweight (BMI 25.0–29.9 kg/m^2^) 7.0 to 11.5 kg; and women with obesity (BMI ≥ 30 kg/m^2^) 5 to 9 kg. Weight gain beneath or over these ranges was considered as inadequate or excessive, respectively [20].

Information on daily calcium intake from food and supplement sources during pregnancy was collected. Calcium intake from foods was evaluated using a semi-quantitative Food Frequency Questionnaire (FFQ) validated for the Greek population [21]. The frequency of consumption of calcium-rich foods was determined as the number of times a food was consumed within a month, in small, medium, or large portion sizes. During the interviews, portion food models and pictures were demonstrated to facilitate the estimation of portion sizes. Dietary calcium intake was quantified with 48 h recall records using the software package Nutritionist Pro™ v7.9 (Axxya Systems, Woodinville, WA, USA). In order to assess consumption of commercially available foods that were not listed in the software, nutritional labels on food packaging were registered in the software package. Detailed information on the consumed supplements was also collected for each participant (type, brand, duration, frequency, and quantity consumed). According to the IOM, the dietary reference intake for calcium during pregnancy (age group 19–50) is 1000 mg daily [22].

### 2.4. Preterm Neonates

Weight and length of each neonate were recorded at birth and z-scores were calculated using the “Fenton Growth Chart” [23]. A weight balance with a sensibility of one gram (Incubator 8000 SCVR, Drager-or-Tassinari Bilance, Milano, Italy) was used to measure birth weight. Total length was measured with a measuring tape to the nearest millimeter.

### 2.5. Blood Measurements

Standard blood withdrawal was performed for each neonate on the first day of life. To collect serum, whole blood was previously allowed to clot at room temperature for 20 min. Whole blood samples were centrifuged at 3000 rpm for 10 min at 4 °C. Serum samples were then stored at −80 °C until analysis. Serum calcium concentrations were quantified in the integrated clinical chemistry and immunoassay analyzer, Dimension^®^ EXL™ 200 Integrated Chemistry System (Siemens Healthcare GmbH, Erlangen, Germany) using manufacturer’s reagents. Hypocalcemia was defined as (a) total serum calcium < 8 mg/dL for preterm infants weighing > 1500 g at birth, and (b) total serum calcium < 7 mg/dL for very low birth weight infants weighing < 1500 g [17].

Measurement of 25-OH-D in neonates was also performed on the first day of life and on the same day for mothers. After blood sampling, 1 mL of sample was centrifuged and serum 25-OH-D was measured by electrochemiluminescence immunoassay “ELCIA” (Roche Diagnostics GmbA, Mannheim, Germany). The role of serum 25-OH-D as a marker of vitamin D status has been extensively discussed in the IOM 2011 report [22]. In fact, deficiency of 25-OH-D in adults was defined as circulating 25-OH-D < 20 ng/mL, inadequacy defined as 20 to 30 ng/mL, and adequacy defined as ≥30 ng/mL [22]. With regard to preterm or full-term infants, there are no recommendations for the routine screening of 25-OH-D levels [24]. However, the minimum concentration considered as beneficial for health is 20 ng/mL, while inadequate levels are <20 ng/mL [25,26].

### 2.6. Statistical Analysis

Statistical analysis was performed using the SPSS software for Windows (Version 21, Armork, NY, USA, IBM Corporation). Significance level was set at *p* < 0.05. The Shapiro–Wilk test was used to assess the normality of variable distribution. Dichotomous variables are presented as counts (N) or relative frequencies (N %), whereas continuous variables are presented as mean and standard variation of the mean (SD). We performed the following tests: (A) One-sample *t*-test to compare neonates’ serum calcium levels with the optimal serum calcium as established in the bibliography [17]. (B) Independent *t*-test to compare serum calcium levels between the following: (i) neonates whose mothers consumed calcium supplements and neonates whose mothers did not. For this purpose, women were categorized into two subgroups: a group not using calcium supplementation and a group receiving 500 mg of calcium supplement per day; and (ii) neonates whose mothers had normal weight and neonates whose mothers had overweight/obesity before pregnancy. (C) Pearson’s or Spearman coefficients (for not normally distributed variables) to identify possible correlations between tested variables. (D) Linear regression analysis with unadjusted and adjusted models to confounders (i.e., adjusted model 1: mothers’ age, pre-pregnancy BMI, and gestational age; adjusted model 2: age, pre-pregnancy BMI, gestational age, and preterm birth weight) in order to investigate the association of neonates’ serum calcium levels with mothers’ calcium supplementation.

To this end, a minimum sample size of 32 pregnant women (16 women receiving calcium supplementation vs. 16 women without calcium supplementation) was sufficient to result in a significant difference of 0.5 mg/dL in neonates’ serum calcium levels between the two groups (SD = 1 using a two-tailed *t*-test with 80% power and a 5% level of significance).

## 3. Results

### 3.1. Enrolled Mothers and Preterm Neonates

**Figure 1:**
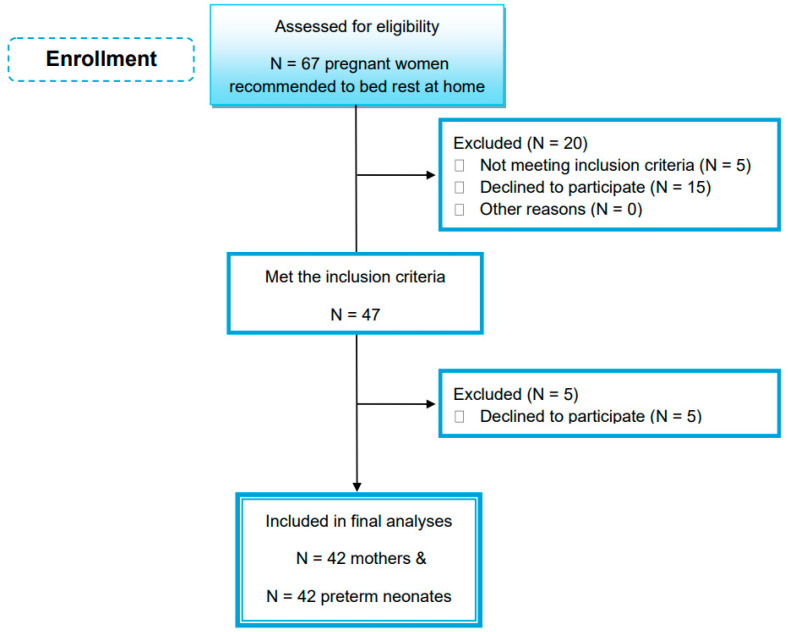
Study flow chart.

As shown in Figure 1, a total of 47 women (out of 67) were eligible and met the inclusion criteria. However, five women withdrew from the study due to personal reasons. As a result, 42 preterm neonates, 16 males and 26 females, and 42 bedridden women were included in the study analysis. All volunteers were Greek, lived in an urban area (Attica) and reported a middle-class socioeconomic status.

### 3.2. Mothers’ Characteristics

Descriptive and clinical characteristics of the enrolled mothers are presented in Table 1. The mean age was 37.55 years, and three out of forty-two mothers were smoking during pregnancy. The mean pre-pregnancy BMI was 23.66 kg/m^2^ and the assessment of pre-pregnancy weight status showed that 2.3% were underweight, 14.4% were overweight, and 7.1% were obese, respectively. The mean BW gain during gestation was 13.45 kg, with 50% of mothers gaining a suboptimal amount of weight and 25% gaining excessive weight.

Mothers with excessive BW gain during gestation demonstrated a significantly higher labor weight compared to mothers of inadequate weight gain (85.90 ± 12.99 vs. 70.27 ± 9.67 kg, respectively, *p* < 0.001) or normal weight gain (85.90 ± 12.99 vs. 72.20 ± 5.29 kg, respectively, *p* = 0.01). Additionally, women with excessive BW gain demonstrated higher post-labor weight than those with inadequate weight gain (75.05 ± 11.0 vs. 65.25 ± 9.6, respectively, *p* = 0.022).

All mothers took dietary supplements during pregnancy. More precisely, the majority of mothers (45.2%) took a combination of nutrients such as calcium-free multivitamins, vitamin D, folic acid, magnesium, and iron.

Additionally, half of the mothers took 500 mg of calcium supplement. Mean daily calcium intake solely from foods was 701.71 mg, ranging from 170 to 1200 mg, and the mean total calcium intake, from both foods and supplements, was 974.33 mg, ranging from 190 to 1535 mg (Table 1). In fact, the mean calcium intake from foods was significantly lower compared to the daily required amount of 1000 mg (*p* < 0.001) [22], with only 11.9% of the mothers meeting this nutrient goal solely through food consumption. Conversely, total calcium intake, from both foods and supplements, was close to the reference intake value (*p* = 0.648). From all mothers, 22 women (52.4%) covered the daily dietary reference intakes for calcium (i.e., 1000 mg) [22], of whom 77.3% used calcium supplements.

The mean value of mothers’ vitamin D serum levels indicated that levels at delivery were insufficient (Table 1). More precisely, 66.7% of pregnant women had serum vitamin D levels below 20 ng/mL, which is considered as vitamin D-deficient, and 9.5% demonstrated adequate levels (equal or greater than 30 ng/mL) [22].

As shown in Supplementary Table S1, the mean total daily calcium intake, from both foods and supplements, significantly differed in mothers who were defined as normal weight based on pre-pregnancy BMI compared to those with overweight or obesity (*p* < 0.001). For underweight women, analysis was not performed, as there was only one participant included. Total calcium intake was significantly higher in mothers who consumed calcium supplements than those who did not (1221.52 ± 204.69 vs. 727.14 ± 313.63 mg/day, respectively, *p* < 0.001).

### 3.3. Characteristics and Calcium Status of Preterm Neonates

Characteristics of preterm neonates are presented in Supplementary Table S2. More specifically, the mean weight and height growth percentiles of neonates from mothers who were categorized as overweight or obese using pre-pregnancy BMI was greater compared to neonates from normal weight mothers (*p* < 0.001). Mean serum calcium of infants was higher than the threshold of hypocalcemia, namely 8 mg/dL for infants weighing > 1500 g (*p* < 0.001) and 7 mg/dL for those weighing ≤ 1500 g (*p* < 0.001). Hypocalcemia was present in four out of forty-two neonates (9.5%). The mean vitamin D serum level was below the threshold value of 20 ng/mL [25,26], and only 14.3% of preterm infants demonstrated adequate serum levels (equal or greater than 20 ng/mL). A strong and statistically significant correlation between neonates’ and mothers’ serum vitamin D levels was found (r = 0.891, *p* < 0.001).

As presented in Table 2, neonates’ serum calcium levels were significantly higher in mothers who used calcium supplementation than those who did not (*p* < 0.001). Additionally, mothers’ total calcium intake correlated positively with the serum calcium levels of neonates (r = 0.414, *p* < 0.001).

To quantify the association of neonates’ calcium status with mothers’ calcium supplement intake, regression analysis was performed (Table 3). Unadjusted regression analysis showed a statistically significant association of neonates’ serum calcium levels with mothers’ calcium supplementation (*p* = 0.023). Upon adjustment for confounders of model 1 (i.e., mothers’ age, pre-pregnancy BMI, and gestational age), this association persisted (*p* = 0.025). A significant association was also evident when neonates’ birth weight was added to the adjusted model (*p* = 0.025).

## 4. Discussion

The adequacy of maternal calcium is undeniably essential for the fetus development, while bed rest during pregnancy is associated with altered markers of bone turnover in pregnant and postpartum women [27]. To the best of our knowledge, there are no literature data available referring to calcium levels of preterm neonates born from bedridden mothers. Therefore, the primary objective of the present study was to assess serum calcium concentration of preterm neonates on the first day of life, and to ascertain its associations with the daily calcium intake from foods and supplements by bedridden pregnant mothers.

We assessed calcium intake during pregnancy based on food or supplement origin. The mean dietary calcium intake was about 30% lower than the daily required amount of 1000 mg, and almost 12 to 100 women met this nutrient goal solely through food consumption. This outcome is in agreement with previous investigations. For instance, results from a large cohort study showed that 42% of pregnant women had a daily calcium intake greater than 800 mg [28]. When calcium supplementation was taken into account in our study, mothers’ total daily calcium intake was close to the daily requirement of 1000 mg/day. In line with this, Willemse and co-authors reported mean calcium intakes from food and supplements equal to 798 mg and 120 mg, respectively [28]. Thus, food sources alone can lead to unsatisfactory calcium intake in pregnant women.

Calcium supplementation is recommended by WHO in order to maintain maternal calcium balance and to support fetal needs, especially in women with low dietary calcium intake (<1000 mg/day) [29]. In our study, total calcium intake (including food and supplement sources) was significantly higher among mothers who took a calcium supplement during pregnancy compared to those who did not. Therefore, supplementation of enrolled pregnant mothers helped them to meet their nutrient requirements. The effect of calcium supplementation during pregnancy on infant outcomes is undoubtful [30]. In the present study, total daily calcium intake from bedridden mothers was positively correlated with neonates’ calcium serum levels. In fact, a higher calcium concentration at birth was associated with mothers receiving a calcium supplement during pregnancy, and this association remained significant after adjustment for mothers’ age, pre-pregnancy BMI, gestational age, and neonates’ birth weight.

Dokos et al. in 2017 reported a mean calcium serum level equal to 8.97 mg/dL in a sample of 20 preterm neonates in Greece [31], a value that is close to the findings of the present study. We also found that four out of forty-two preterm neonates demonstrated low calcium serum levels, indicating hypocalcemia. Hypocalcemia is a common metabolic abnormality in infants, with vitamin D playing a pivotal role in calcium homeostasis [18]. Hypocalcemia may have later impacts on bone mineralization and formation, and on nerves and muscle function. What is more, poor bone mineralization can compromise pulmonary status and growth [18]. Vitamin D (25-OH-D) deficiency is a major health problem globally and, when accompanied by poor calcium intake, is responsible for developing nutritional rickets and osteomalacia [32]. In a recent retrospective study, 48.9% of neonates born by high-risk mothers for 25-OH-D deficiency demonstrated inadequate levels of 25-OH-D [32].

It is well-confirmed that the compensatory mechanisms for calcium maintenance in neonates born by vitamin D-deficient mothers can be depleted quickly, leading to hypocalcemia [33]. In kidneys, the parathyroid hormone stimulates osteoclasts to increase bone resorption and maintain blood calcium levels, while the impaired renal phosphate absorption and the decreased phosphate levels may lead to nutritional rickets and osteomalacia [33]. According to a previously published work, the high prevalence of 25-OH-D deficiency in bedridden pregnant women was probably attributed to insufficient exposure to sunlight and a low dietary vitamin D intake. At the same time, an increase in mothers’ 25-OH-D serum levels during gestation was associated with an increased possibility that the preterm neonates would be measured to have normal 25-OH-D levels [34]. Similarly, in the present study, only 14.3% of preterm infants born by bedridden mothers demonstrated adequate serum 25-OH-D levels (≥20 ng/mL), while a strong and statistically significant correlation between neonates’ and mothers’ serum vitamin D levels was found.

It is well-known that pre-pregnancy overweight and obesity is negatively associated with diet quality during pregnancy, including micronutrient intake [35]. According to population-based studies, a low calcium intake is evident in pregnant women with overweight and obesity [36,37], increasing the risk for pre-eclampsia [38]. The negative associations of dietary calcium with fasting glucose levels and markers of chronic inflammation such as C-reactive protein (CRP) may explain the protective role of calcium in obesity pathogenesis [39]. In line with these observations, our study showed that pregnant women with overweight/obesity who were recommended bed rest consumed less calcium from food and supplements than their normal weight counterparts.

Calcium homeostasis in the newborn could also be affected by mothers’ weight status. There is some evidence that the increased subcutaneous fat in adults abates cutaneous synthesized D3, the precursor of 25-OH-D [40]. To this end, Bodnar and co-workers showed that there is a dose-response trend between pre-pregnancy obesity and vitamin D deficiency in the newborn affecting calcium homeostasis [41]. In the present study, we measured calcium concentration in preterm neonates based on mothers’ pre-pregnancy weight status, namely normal weight and overweight/obesity. Despite the fact that the total calcium intake was greater in normal weight mothers, no significant differences were found for neonates’ calcium levels between the two groups. This observation could be attributed to the relatively low sample size of our study, as well as to the complexity of the altered endocrine system during pregnancy in the presence of obesity [41]. All in all, the results of our study indicate that pregnant women who were recommended bed rest could benefit from calcium supplementation. The observed significant association between mothers’ calcium intake and infants’ calcium status supports this hypothesis and implies that adequate calcium intake during pregnancy is critical for maintaining infants’ bone health. This is further endorsed by the observation that neonates’ 25-OH-D levels were strongly correlated with the mothers’ status of 25-OH-D, a key molecule in calcium metabolism and bone development [13]. However, larger prospective case-control studies are needed to identify the role of calcium supplementation by bedridden mothers, with or without the presence of overweight/obesity, in preterm health outcomes.

Our study has some limitations. We recognize that the sample size of the study is relatively small. What is more, calcium homeostasis in neonates may be influenced by additional factors, e.g., parathyroid hormone, calcitonin, phosphate levels, that were not evaluated in this study. Nevertheless, to the best of our knowledge, the present study was the first to investigate the association of neonates’ calcium status with bedridden mothers’ calcium intake, from either foods or supplements. In the future, large prospective case-control studies are needed to explore the effects of calcium supplementation by bedridden mothers on calcium homeostasis, as well as the growth and development of preterm infants. We are also aware that the collection of self-reported data regarding calcium intake could be a source of bias. To minimize such bias, the FFQ used in the present study was already validated in the Greek population. Additionally, the appointed dieticians were well-experienced, being able to resolve possible disparities detected in FFQ and dietary recall records.

## 5. Conclusions

In the present study, hypocalcemia was observed as an occurrence in preterm neonates. Bedridden pregnant women who had a premature delivery benefited from calcium supplementation compared to those who did not. In fact, mothers taking calcium supplementation met the recommended daily reference intake for calcium, and total calcium intake correlated significantly with neonates’ serum calcium levels. Additionally, mothers’ serum vitamin D levels correlated significantly with neonates’ vitamin D status. Studies with an increased number of enrolled mothers (bedridden and non-bedridden) and neonates could further help to clarify the influences and the role of certain nutrient intake during pregnancy in neonatal outcomes.

## Figures and Tables

**Table 1:** Characteristics of enrolled mothers.

Mothers’ Characteristics	N (%)	Mean ± SD
Age at delivery (years)	42 (100)	37.55 ± 6.74
Smoking status		
Smoker	3 (7.1)	-
Non-smoker	39 (92.9)	-
Reasons for bed rest		
Placenta abruption	40 (95.2)	-
Short cervical length	2 (4.8)	-
Duration of bed rest (months)	42 (100)	2.26 ± 0.33
Pre-pregnancy BMI (kg/m^2^)	42 (100)	23.66 ± 3.95
Underweight (BMI < 18.5 kg/m^2^)	1 (2.4)	-
Normal weight (BMI = 18.5–24.9 kg/m^2^)	32 (76.2)	22.14 ± 1.76
Overweight (BMI = 25–29.9 kg/m^2^)	6 (14.3)	27.85 ± 1.91
Obesity (BMI ≥ 30 kg/m^2^)	3 (7.1)	33.56 ± 2.74
Body weight gain during pregnancy (kg)	42 (100)	13.45 ± 8.27
Inadequate	22 (52.4)	9.05 ± 4.46
Normal	10 (23.8)	15.20 ± 6.25
Excessive	10 (23.8)	21.40 ± 10.27
Body weight on the day of delivery (kg)	42 (100)	74.45 ± 11.56
Body weight on the day after delivery (kg)	42 (100)	68.24 ± 9.75
Serum 25-OH-D levels (ng/ml)	42 (100)	17.24 ± 10.07
Serum 25-OH-D status		
Adequacy (≥30 ng/mL)	4 (9.5)	37.55 ± 10.36
Inadequacy (20–29 ng/mL)	10 (23.8)	24.93 ± 2.79
Deficiency (<20 ng/mL)	28 (66.7)	11.60 ± 4.85
Women taking oral supplements other than calcium		
Prenatal multivitamin	10 (23.8)	-
Vitamins (D, folic acid)	7 (16.7)	-
Minerals (Fe, Mg)	6 (14.3)	-
Combination of prenatal multivitamins, vitamins (D, folic acid) and minerals (Fe, Mg)	19 (45.2)	-
Daily intake of calcium supplement during pregnancy		
Use of 500 mg calcium supplement /day	21 (50.0)	-
No use of calcium supplement	21 (50.0)	-
Total daily calcium intake from foods and supplements during pregnancy (mg/day) in all women	42 (100)	974.33 ± 361.96
Total daily calcium intake during pregnancy		
Equal or greater than 1000 mg/day	22 (52.4)	1258.45 ± 145.01
Lower than 1000 mg/day	20 (47.6)	661.80 ± 251.37
Daily calcium intake solely from foods during pregnancy (mg/day) in all women	42 (100)	701.71 ± 263.12
Daily calcium intake solely from foods during pregnancy		
≥1000 mg of food derived calcium /day	5 (11.9)	1112.00 ± 69.96
<1000 mg of food derived calcium /day	37 (88.1)	646.27 ± 227.51

Data are presented as counts (N), relative frequencies (N %), or mean ± standard deviation of mean (SD).

**Table 2:** Neonates’ serum calcium levels based on mothers’ pre-pregnancy weight statuses and calcium supplementation.

Neonates’ Serum Calcium Levels (mg/dL) (N = 42)	N (N %)	Mean ± SD	*p*-Value
Based on mothers’ pre-pregnancy BMI status			
Normal weight	32 (76.2)	8.47 ± 0.80	0.143
Overweight/obesity	9 (21.4)	8.92 ± 0.80	
Based on mothers’ calcium supplementation			
Calcium supplementation group	21 (50.0)	8.90 ± 0.58	<0.001 *
Non-calcium supplementation group	21 (50.0)	8.31 ± 0.97	

Data are presented as counts (N), relative frequencies (N %), or mean ± standard deviation of mean (SD). * *p*-value: Independent *t*-test comparisons for serum calcium levels between neonates whose mothers consumed calcium supplements (500 mg calcium/day) and neonates whose mothers did not; significant difference was set at *p* < 0.05.

**Table 3:** Regression analysis.

Tested Association	Unadjusted Model		Adjusted Model 1		Adjusted Model 2	
Neonates’ Ca status	Beta	*p*-value	Beta	*p*-value	Beta	*p*-value
Mother’s Ca supplementation	+0.351	0.023	+0.360	0.025	+0.460	0.025

Level of statistical significance was set at *p* < 0.05. *p*-value represents statistically significant associations between variables. Model 1: adjusted for mothers’ age, pre-pregnancy BMI, and gestational age. Model 2: adjusted for mothers’ age, pre-pregnancy BMI, gestational age, and neonates’ birth weight.

## Data Availability

Data are contained within the article and Supplementary Materials.

## Supplementary Materials

The following supporting information can be downloaded at https://www.mdpi.com/article/10.3390/healthcare12060693/s1, Table S1: Daily calcium intake by mothers based on pre-pregnancy weight status and calcium supplementation; Table S2: Characteristics of preterm neonates.
