# Supplementary material for: Association of Serum Calcium Levels of Preterm Neonates at Birth with Calcium Intake from Foods and Supplements by Bedridden Women during Pregnancy
Source: Healthcare (Basel). 2024 Mar 20;12(6):693. doi: 10.3390/healthcare12060693 (PMC10970113; doi:10.3390/healthcare12060693)
Supplement: Supplementary file 1 [file healthcare-12-00693-s001.zip › healthcare-2901788-supplementary.pdf]

Supplementary Materials

Association of Serum Calcium Levels of Preterm Neonates at Birth with Calcium Intake from Foods and Supplements by Bedridden Women during Pregnancy

Supplementary Table S1. Daily calcium intake by mothers based on pre-pregnancy weight status and calcium supplementation.

**Mothers' total daily calcium intake during pregnancy (mg/day)**	**N (N %)**	**Mean ± SD**	***p-value***
Based on the pre-pregnancy BMI status			< 0.001*
Normal weight	32 (76.2)	979.72 ± 360.15	
Overweight / obesity	9 (21.4)	922.78 ± 393.83	
Based on mothers' calcium supplementation			< 0.001**
Calcium supplementation group	21 (50.0)	1221.52 ± 204.69	
Non-calcium supplementation group	21 (50.0)	727.14 ± 313.63	

Data are presented as counts (N), relative frequencies (N %) or mean ± standard deviation of mean (SD). **p-value:* Independent t-test comparisons for total daily calcium intake between mothers with normal weight and mothers with overweight / obesity based on pre-pregnancy BMI; significant difference was set at $p < 0.05$. ***p-value:* Independent t-test comparisons for total daily calcium intake between mothers who consumed calcium supplements (500 mg calcium /day) during pregnancy and mothers who did not; significant difference was set at $p < 0.05$.

Supplementary Table S2. Characteristics of preterm neonates.

**Characteristics of preterm neonates**	**N (N %)**	**Mean ± SD**	***p-value***
Gestational age (weeks)	42 (100)	29.33 ± 1.78	-
Total length (cm)	42 (100)	38.81 ± 2.90	-
Birth weight (g)	42 (100)	1214.88 ± 283.90	-
Birth weight ≤ 1500 g	35 (83.3)	1137.86 ± 244.45	-
Birth weight > 1500 g	7 (16.7)	1600.00 ± 67.08	-
Z score length for age	42 (100)	0.46 ± 0.88	-
Percentiles length for age	42 (100)	62.83 ± 22.69	-
Z score weight for age	42 (100)	-0.11 ± 0.73	-
Percentiles weight for age	42 (100)	44.10 ± 19.85	-
Serum calcium levels (mg/dL)	42 (100)	8.61 ± 0.84	-
Serum 25-OH-D levels (ng/ml)	42 (100)	12.02 ± 6.82	-
Adequacy (≥ 20 ng/mL)	6 (14.3)	24.63 ± 2.01	-
Insufficiency (< 20 ng/mL)	36 (85.7)	9.92 ± 4.70	-
Serum calcium levels based on birth-weight cutoff (mg/dL)			
Birth weight ≤ 1500 g	35 (83.3)	8.76 ± 0.76	< 0.001*
Birth weight > 1500 g	7 (16.7)	7.83 ± 0.87	
Percentiles weight for age based on mothers' pre-pregnancy BMI status			
Normal weight	32 (76.2)	40.28 ± 17.51	
Overweight / obesity	9 (21.4)	59.33 ± 21.9	< 0.001**
Percentiles length for age based on mothers' pre-pregnancy BMI status			
Normal weight	32 (76.2)	60.88 ± 21.11	
Overweight / obesity	9 (21.4)	70.22 ± 28.83	< 0.001**

Data are presented as counts (N), relative frequencies (N %) or mean ± standard deviation of mean (SD). **p-value:* Independent t-test comparisons for serum calcium levels between neonates at birth weight ≤ 1500 and those at > 1500 g; significant difference was set at $p < 0.05$. ***p-value:* Independent t-test comparisons for neonates' body weight and height growth percentiles between mothers with normal weight and those with pre-pregnancy overweight / obesity; significant difference was set at $p < 0.05$.
